# Is GFR decline induced by SGLT2 inhibitor of clinical importance?

**DOI:** 10.1186/s12933-024-02223-0

**Published:** 2024-05-29

**Authors:** Merve Günes-Altan, Agnes Bosch, Kristina Striepe, Peter Bramlage, Mario Schiffer, Roland E. Schmieder, Dennis Kannenkeril

**Affiliations:** 1 Department of Nephrology and Hypertension, University Hospital Erlangen Friedrich-Alexander University Erlangen-Nürnberg (FAU), Ulmenweg 18, 91054 Erlangen, Germany; 2 Institute for Pharmacology and Preventive Medicine, Bahnhofstraße 20, 49661 Cloppenburg, Germany; 3 Department of Cardiology, University Hospital Erlangen, Friedrich-Alexander University Erlangen-Nürnnberg (FAU), Ulmenweg 18, 91054, Erlangen, Germany

**Keywords:** GFR decline, PWV, SGLT2 inhibitors

## Abstract

**Background:**

Use of sodium-glucose-cotransporter-2 (SGLT2) inhibitors often causes an initial decline in glomerular filtration rate (GFR). This study addresses the question whether the initial decline of renal function with SGLT2 inhibitor treatment is related to vascular changes in the systemic circulation.

**Methods:**

We measured GFR (mGFR) and estimated GFR (eGFR) in 65 patients with type 2 diabetes (T2D) at baseline and after 12 weeks of treatment randomized either to a combination of empagliflozin and linagliptin (SGLT2 inhibitor based treatment group) (*n* = 34) or metformin and insulin (non-SGLT2 inhibitor based treatment group) (*n* = 31). mGFR was measured using the gold standard clearance technique by constant infusion of inulin. In addition to blood pressure (BP), we measured pulse wave velocity (PWV) under standardized conditions reflecting vascular compliance of large arteries, as PWV is considered to be one of the most reliable vascular parameter of cardiovascular (CV) prognosis.

**Results:**

Both mGFR and eGFR decreased significantly after initiating treatment, but no correlation was found between change in mGFR and change in eGFR in either treatment group (SGLT2 inhibitor based treatment group: *r*=-0.148, *p* = 0.404; non-SGLT2 inhibitor based treatment group: *r* = 0.138, *p* = 0.460). Noticeably, change in mGFR correlated with change in PWV (*r* = 0.476, *p* = 0.005) in the SGLT2 inhibitor based treatment group only and remained significant after adjustment for the change in systolic BP and the change in heart rate (*r* = 0.422, *p* = 0.018). No such correlation was observed between the change in eGFR and the change in PWV in either treatment group.

**Conclusions:**

Our main finding is that after initiating a SGLT2 inhibitor based therapy an exaggerated decline in mGFR was related with improved vascular compliance of large arteries reflecting the pharmacologic effects of SGLT2 inhibitor in the renal and systemic vascular bed. Second, in a single patient with T2D, eGFR may not be an appropriate parameter to assess the true change of renal function after receiving SGLT2 inhibitor based therapy.

**Trial registration:**

clinicaltrials.gov (NCT02752113).

## Introduction

Sodium-glucose-cotransporter-2 (SGLT2) inhibitors have demonstrated remarkable cardiovascular (CV) and renal benefits beyond glycemic control in several trials and emerged as therapeutic agents in the treatment of heart failure and chronic kidney disease [1–10]. However, it has been repeatedly described that initiation of SGLT2 inhibitor therapy causes an initial decline of estimated glomerular filtration rate (eGFR), which may lead to physician’s concern [1, 6, 7, 11–15]. This initial decline in eGFR with SGLT2 inhibitor treatment is suggested to be related to a reduction in intraglomerular pressure [7, 11–16]. The mechanism underlying the reduction of the intraglomerular pressure with SGLT2 inhibitor treatment appears to be different in different cohorts. Previously, we showed in type 2 diabetes (T2D) patients with empagliflozin treatment that this reduction in intraglomerular pressure is caused by a postglomerular vasodilatation, rather than a preglomerular vasoconstriction by tubuloglomerular feedback as demonstrated in patients with type 1 diabetes and experimental studies [16–18]. In support to our study, van Bommel et al. observed similar results in T2D patients with dapagliflozin treatment [19]. Although this vasodilatory effect on the efferent arterioles results in an initial drop in eGFR, in the long term it comes to an improvement of kidney function and slows the progression of kidney disease [1, 3, 6, 7]. 

The predictive and clinical value of the initial eGFR decline remains controversial. While the post-hoc analysis of the EMPA-REG OUTCOME trial did not find any association between the initial “eGFR dip” and the treatment effect of empagliflozin, similar analysis of the DAPA-HF trial showed better clinical outcome in patients with initial eGFR decline after dapagliflozin treatment [11, 14]. In consequence, this controversial results regarding initial decline in eGFR might result in an inappropriate discontinuation of the pharmacotherapy by healthcare providers or patients themselves, out of fear that the medication could harm the kidneys. This may result in a missed opportunity to slow the progression of kidney and CV disease. Thus, there is a crucial need to understand the implications of the initial GFR decline after the beginning of SGLT2 inhibitor treatment.

Previously, we have shown that SGLT2 inhibitor treatment exerts beneficial effects on different vascular parameters, e.g. in the renal and systemic circulation in different cohorts [20–22]. The main aim of the current study was to evaluate whether there is a relationship between the initial GFR decline assessed using input steady state input clearance technique with inulin and vascular changes in the systemic circulation. In addition to renal function, we measured pulse wave velocity (PWV) since according to several guidelines [23–25] PWV is recommended to most reliably reflect vascular compliance of large arteries in the systemic circulation. Moreover, PWV has been identified to be an independent prognostic marker of CV fatal events [26–30] and its change to improved prognosis [31]. 

## Materials and methods

### Study design

This single-center, retrospective study includes 65 patients with T2D who were randomized (1:1) either to receive empagliflozin and linagliptin (SGLT2 inhibitor based treatment) or metformin and insulin (non-SGLT2 inhibitor based treatment) combination therapy. We analyzed the SGLT2 inhibitor based treatment group and the non-SGLT2 inhibitor based treatment group (control group) separately. The rationale for these combination treatments was to compare the renal hemodynamic effects of the timely standard combination T2D treatment (SGLT2 inhibitor + Dipeptidyl peptidase 4 inhibitor) with the combination of insulin and metformin (traditional or old way of treatment). All patients participated in the „Effects of Empagliflozin + Linagliptin vs. Metformin + Insulin Glargine on Renal and Vascular Changes in Type 2 Diabetes (ELMI)“ trial (NCT02752113) between April 2016 and November 2018 in our clinical research center at the University Hospital Erlangen-Nuremberg (www.crc-erlangen.de). The main results of the effects of these two different anti-hyperglycmic treatment strategies have been reported previously [17, 20]. Here we analyzed the prespecified analysis of patients with completed inulin clearance at baseline and after 12 weeks of treatment (The inulin application had to be immediately stopped in our lab after an official warning due to anaphylactic reactions observed during infusions of inulin in France, which ultimately lead to the withdrawal of inulin from the market).

All patients had stable metformin medication for at least 3 months (850 or 1000 mg orally twice daily). Patients that were randomized to the SGLT2inhibitor based treatment study arm received 10 mg empagliflozin and 5 mg linagliptin orally once daily. Empagliflozin was uptitrated to 25 mg if fasting blood glucose was ≥ 100 mg/dl. Patients in the non-SGLT2 inhibitor based treatment group received initially 2–4 units (U) of insulin subcutaneous once daily in addition to their metformin medication. It was adjusted every third day by adding 2 U if fasting blood glucose was not ≤ 125 mg/dl until stable dose was reached.

The respective study was approved by the local Ethical Review Committee (ethics committee of the University of Erlangen-Nuremberg, Germany) and the study was conducted according to the Declaration of Helsinki. Written informed consent was obtained from all patients prior to study inclusion.

### Study cohort

A total of 65 patients with T2D aged 18–75 were included in this analysis. Thirty-four patients have been allocated to the SGLT2 inhibitor based treatment group and 31 to the non-SGLT2 inhibitor based treatment group. The eligibility criteria for the study included an glycated haemoglobin (HbA1c) level of ≥ 6.5% for those individuals with antidiabetic monotherapy and ≥ 6.0% for those receiving dual antidiabetic therapy. Main exclusion criteria were the use of insulin, glitazones, dipeptidyl peptidase-4 inhibitor or SGLT2 inhibitor within two months prior to randomization. Patients with congestive heart failure New York Heart Association (NYHA) III/IV were excluded. Furthermore, patients with HbA1c > 10.5% or fasting plasma glucose > 240 mg/dl, urinary albumin to creatinine ratio (UACR) > 300 mg/g, eGFR < 60 ml/min/1.73 m², body-mass-index (BMI) > 40 kg/m² or cardio- and cerebro-vasacular events within the past 6 months were not considered for the study. For female patients, a negative pregnancy test was mandatory before and during the study period.

### Assessments

Vascular and renal function measurements were performed at baseline and after 12 weeks of treatment. These measurements have been described in detail previously [17, 20]. Briefly, before attended BP measurement was performed the patient remained seated and relaxed for 3–5 min. An appropriate cuff size was selected according to the arm circumference of each individual. Office BP were calculated from the average of three measurements and were measured with validated devices following the recommendations of the European Society of Hypertension/European Society of Cardiology [32, 33]. . The SphygmoCor™ system (AtCor Medical, Sydney, Australia) was used for the vascular assessment of the compliance of large arteries by measuring pulse wave velocity (PWV) under standardized conditions in our research facility.

Constant-infusion input-clearance technique with inulin (Inutest, Fresenius, Linz, Austria) and sodium p-aminohippurate (PAH) (Daiichi Sankyo, Tokyo, Japan) were used to measure the true renal function (mGFR) and renal plasma flow (RPF), respectively. This method is the most widely used method for the measurement of mGFR since a good correlation between the traditional method with urine collection and the constant infusion technique without urine collection (*r* = 0.993) has been demonstrated [34]. Based on serum creatinine measurements, the eGFR was calculated according to the Chronic Kidney Disease Epidemiology Collaboration (CKD-EPI) formula [17]. 

### Statistical analysis

Statistical analysis was performed using SPSS Statistics 28.0 (IBM, Armonk, New York, USA) and data were expressed as mean ± standard deviation (SD) in text and tables. A two-sided *p*-value of < 0.05 was considered statistically significant. Paired t-test was applied for the comparison of the end of 12 weeks treatment phase versus baseline within each treatment group. The unpaired Student’s t-test was used to determine the statistical significance of the differences between the SGLT2 inhibitor based and non-SGLT2 inhibitor based treatment arms. Bivariate correlation analyses were assessed by performing Pearson’s test. The correlation analysis of the parameter UACR was assessed by performing Spearman’s test, since UACR was not normally distributed. Since change of PWV is strongly dependent on change in systolic BP, we adjusted our approach to the change of systolic BP and the change of heart rate after 12 weeks by applying covariance analysis. The Bland-Altman Plot was used as a descriptive tool to evaluate the agreement between two methods, showing the relationship between the change of mGFR and eGFR and the mean of the two methods.

## Results

### Clinical characteristics at baseline and after 12 weeks of treatment

The clinical characteristics of the SGLT2 inhibitor based treatment (*n* = 34) and non-SGLT2 inhibitor based treatment (*n* = 31) groups are shown in Table 1. The average age of the patients was 59.4 ± 8.4 years in the SGLT2 inhibitor based treatment group and 59.9 ± 9.7 years in the non-SGLT2 inhibitor based treatment group. 77% of the patients were male with no between group differences. The two groups did not differ in terms of demographic data.

**Table 1:** Baseline characteristics

Parameters	SGLT2 inhibitor based treatment group (*n* = 34)	Non-SGLT2 inhibitor based treatment group (*n* = 31)	*p*-value
Demographic data			
Age (years)	59.4 ± 8.4	59.9 ± 9.7	0.807
Gender (m/f)	27/7	23/8	0.618
Weight (kg)	90.6 ± 15.1	94.9 ± 18.4	0.306
BMI (kg/m²)	30.2 ± 3.6	31.6 ± 3.8	0.138
**Laboratory values**			
Fasting plasma glucose (mg/dl)	169.1 ± 30.4	170.1 ± 32.8	0.895
HbA1c (%)	7.9 ± 0.7	8.0 ± 0.7	0.803
Total cholesterol (mg/dL)	208.4 ± 39.3	210.6 ± 34.1	0.811
LDL-cholesterol (mg/dL)	136.4 ± 29.6	139.8 ± 26.4	0.635
HDL-cholesterol (mg/dL)	47.7 ± 10.7	45.8 ± 10.1	0.466
Triglycerides (mg/dL)	215.5 ± 120.4	219.0 ± 107.9	0.902
Hs-CRP (mg/L)	2.0 ± 2.9	3.0 ± 2.5	0.158
Hemoglobine (g/dL)	14.5 ± 1.0	14.4 ± 0.9	0.561
Hematocrit (%)	42.6 ± 2.6	42.5 ± 2.8	0.835
Creatinine (mg/dL)	0.8 ± 0.2	0.8 ± 0.2	0.561
eGFR (ml/min/1.73 m²)	94.8 ± 10.6	93.2 ± 10.5	0.547
mGFR (ml/min)	126.5 ± 13.0	126.5 ± 14.8	0.991
**BP**			
Systolic BP (mmHg)	135.1 ± 10.8	134.9 ± 10.3	0.922
Diastolic BP (mmHg)	82.4 ± 8.2	81.9 ± 10.6	0.835
Heart rate (bpm)	74.5 ± 11.8	72.1 ± 11.6	0.406

Data are presented as mean ± standard deviation

BMI – body mass index, HbA1c – glycated hemoglobin, LDL – low density lipid, HDL – high density lipid, hs-CRP – high sensitive C reactive protein, eGFR - estimated glomerular filtration rate, CKD-EPI - Chronic Kidney Disease Epidemiology Collaboration calculated based on CKD-EPI formula, UACR –urine albumin-creatinine ratio (spontaneous urine), mGFR - measured glomerular filtration rate, BP –blood pressure, bpm – beats per minute

We observed a significant reduction of glycaemic parameters like fasting plasma glucose and HbA1c after 12 weeks in both treatment arms (Table 2). Patients in the SGLT2 inhibitor based treatment arm had a significant reduction in weight and BMI, whereas no change in these parameters was observed in the non-SGLT2 inhibitor based treatment group (Table 2). The effects on renal hemodynamics of the SGLT2 inhibitor based treatment group and non-SGLT2 inhibitor based treatment group have been described previously [17] and are presented in Table 2 for the patients included in the current analysis. Renal function decreased in both treatment groups irrespective whether analysed by mGFR or eGFR. Briefly, in patients with SGLT2 inhibitor based treatment, PWV decreased from 8.2 ± 1.6 to 7.8 ± 1.5 m/s after 12 weeks of treatment (*p* = 0.028), whereas no change in PWV occurred after 12 weeks of treatment in the non-SGLT2 inhibitor based treatment group (*p* = 0.169).

**Table 2:** Changes in baseline characteristics after 12 weeks of treatment with SGLT2 inhibitor based therapy or non-SGLT2 inhibitor based treatment (only those variables with *p* < 0.05 are shown)

Parameters	SGLT-2 inhibitor based treatment		*p*-value vs. Baseline	Non-SGLT2 inhibitor based treatment		*p*-value vs. Baseline
	Baseline	12 weeks		Baseline	12 weeks	
Demographic data						
Weight (kg)	90.6 ± 15.1	87.8 ± 14.2	**< 0.001**	94.9 ± 18.4	94.4 ± 18.3	0.247
BMI (kg/m²)	30.1 ± 3.6	29.3 ± 3.4	**< 0.001**	31.5 ± 3.8	31.5 ± 3.9	0.981
**Laboratory values**						
Fasting plasma glucose (mg/dl)	158.4 ± 27.9	138.1 ± 23.6	**< 0.001**	159.8 ± 26.0	123.0 ± 20.2	**< 0.001**
HbA1c (%)	7.7 ± 0.6	7.3 ± 0.7	**< 0.001**	7.8 ± 0.7	6.9 ± 0.8	**< 0.001**
**Blood pressure**						
Systolic BP (mmHg)	135.1 ± 10.8	127.1 ± 10.6	**0.002**	134.9 ± 10.3	127.5 ± 11.7	**0.042**
Diastolic BP (mmHg)	82.4 ± 8.2	77.8 ± 9.2	**0.001**	81.9 ± 10.6	76.7 ± 9.2	**< 0.001**
Heart rate (bpm)	74.5 ± 11.8	69.8 ± 10.2	**< 0.001**	72.1 ± 11.6	70.8 ± 10.3	**< 0.001**
**Renal hemodynamics**						
eGFR (ml/min/1.73 m²)	94.8 ± 10.6	81.6 ± 20.8	**< 0.001**	93.2 ± 10.5	82.1 ± 22.8	**0.002**
mGFR (ml/min)	126.5 ± 13.0	120.2 ± 13.8	**0.003**	126.5 ± 14.8	119.8 ± 13.3	**0.001**
RPF (ml/min)	597.3 ± 108.3	584.1 ± 105.9	0.401	611.8 ± 128.0	568.5 ± 105.6	**0.005**
**Pulse wave velocity**						
PWV (m/s)	8.2 ± 1.6	7.8 ± 1.5	**0.028**	8.5 ± 1.3	8.3 ± 1.1	0.169

Data are presented as mean ± standard deviation

SGLT2- sodium-glucose-cotransporter-2, BMI – Body Mass Index, blood pressure – BP, bpm – beats per minute, eGFR – estimated glomerular filtration rate, mGFR – measured glomerular filtration rate calculated based on CKD-EPI formula, HbA1c – glycated hemoglobin, RPF – renal plasma flow, PWV – pulse wave velocity

**Table 3:** Correlation between change of PWV or hsCRP with the change of mGFR or eGFR **A**: SGLT2 inhibitor based treatment group

	SGLT2 inhibitor based treatment			
	Change of mGFR		Change of eGFR	
	r-value	*p*-value	r-value	*p*-value
**Change of PWV**	0.476	**0.005**	-0.038	0.834
**Change of hsCRP**	0.376	**0.031**	0.061	0.735
**Change of office systolic BP**	0.398	**0.023**	-0.016	0.927
**Change of office diastolic BP**	0.354	**0.040**	-0.023	0.898
**Change of UACR**	0.367	**0.033**	0.011	0.953
**Change of HbA1c**	-0.235	0.181	0.136	0.443
**Change of fasting plasma glucose**	0.177	0.317	0.025	0.889

SGLT2- sodium-glucose-cotransporter-2, eGFR - estimated glomerular filtration rate, CKD-EPI - Chronic Kidney Disease Epidemiology Collaboration calculated based on CKD-EPI formula, mGFR - measured glomerular filtration rate, PWV – pulse wave velocity, BP –blood pressure, UACR –urine albumin-creatinine ratio (spontaneous urine), HbA1c – glycated hemoglobin, hs-CRP – high sensitive C reactive protein

**B:** Non-SGLT2 inhibitor based treatment group

	Non-SGLT2 inhibitor based treatment			
	Change of mGFR		Change of eGFR	
	r-value	*p*-value	r-value	*p*-value
**Change of PWV**	0.078	0.678	0.189	0.308
**Change of hsCRP**	-0.218	0.239	-0.255	0.166
**Change of office systolic BP**	0.175	0.348	0.071	0.703
**Change of office diastolic BP**	-0.021	0.913	0.125	0.502
**Change of UACR**	-0.036	0.851	0.265	0.166
**Change of HbA1c**	0.312	0.099	-0.246	0.199
**Change of fasting plasma glucose**	0.167	0.377	-0.066	0.727

SGLT2- sodium-glucose-cotransporter-2, eGFR - estimated glomerular filtration rate, CKD-Epi - Chronic Kidney Disease Epidemiology Collaboration calculated based on CKD-EPI formula, mGFR - measured glomerular filtration rate, PWV – pulse wave velocity, BP –blood pressure, UACR –urine albumin-creatinine ratio (spontaneous urine), HbA1c – glycated hemoglobin, hs-CRP – high sensitive C reactive protein

### Relationship between renal and systemic vascular bed

**Fig. 1:**
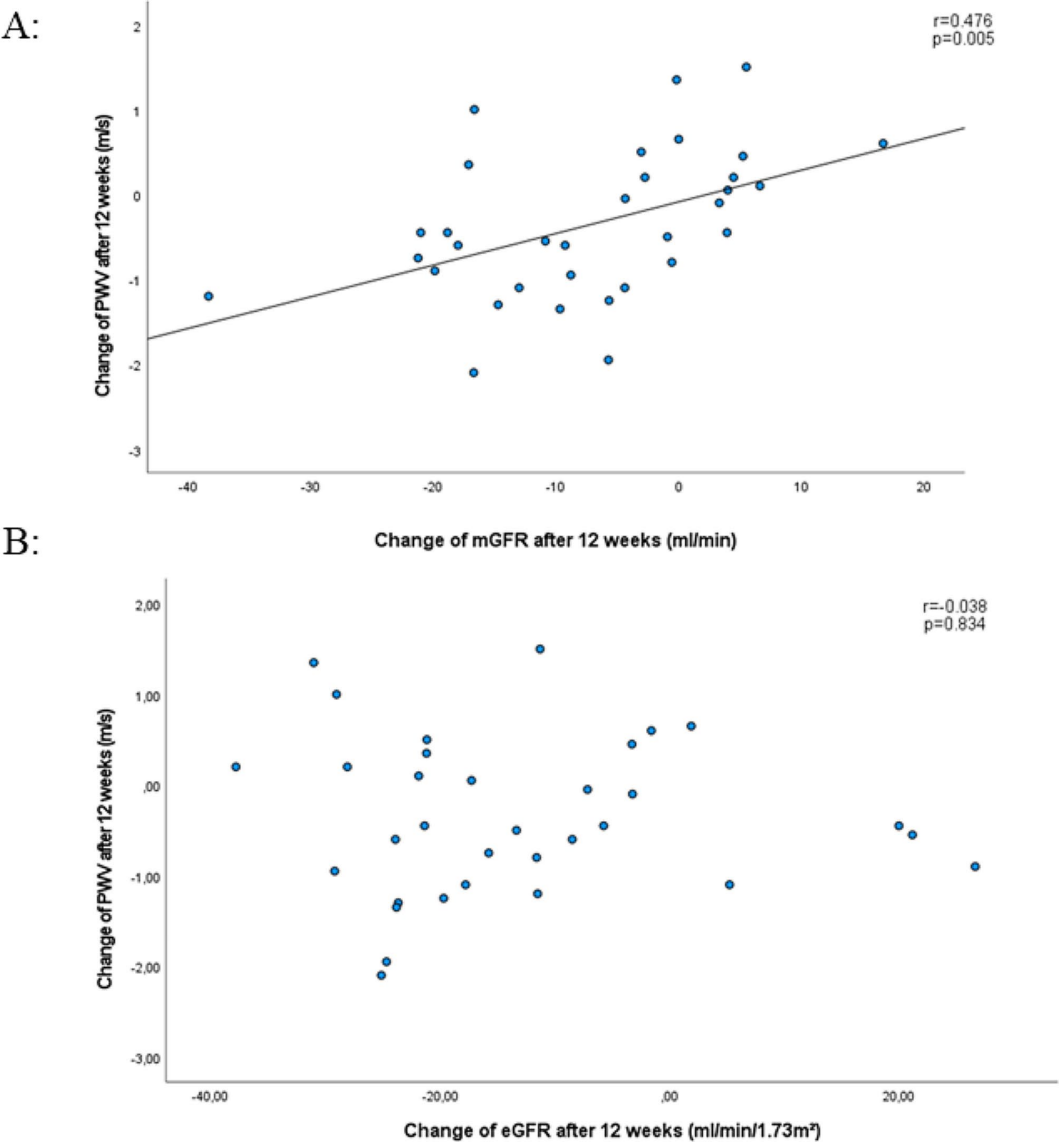
Correlation between change in mGFR (**A**; upper part) or change in eGFR (**B**; lower part) and change in PWV after 12 weeks of treatment in SGLT2 inhibitor based treatment group

**Fig. 2:**
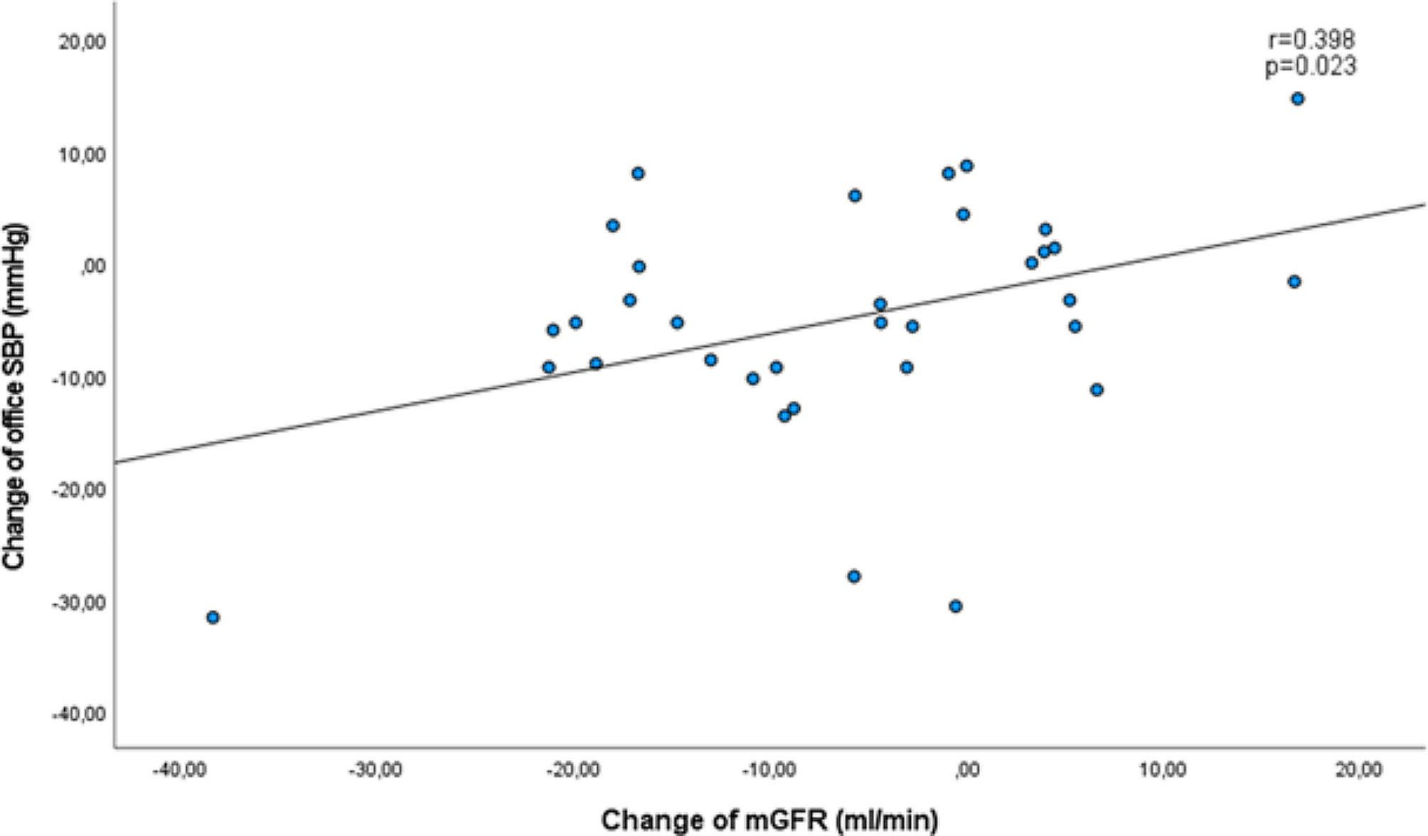
Correlation between change in mGFR and change in office systolic blood pressure after 12 weeks of treatment in SGLT2 inhibitor based treatment group

The change of mGFR in the SGLT2 inhibitor based treatment group correlated with the change of PWV (*r* = 0.476, *p* = 0.005, Table 3A, Fig. 1A). Such a correlation could not be shown between the change of eGFR and the change of PWV (*r*=-0.038, *p* = 0.834, Table 3A, Fig. 1B). We did not found any correlation between PWV and mGFR or eGFR in the non-SGLT2 inhibitor based treatment group (all *p* > 0.10, Table 3B). Similarly, we found a correlation between change of mGFR and change of office systolic BP (*r* = 0.398, *p* = 0.023; Fig. 2) as well as change of office diastolic BP (*r* = 0.354, *p* = 0.040) in the SGLT2 inhibitor based treatment group, but no correlation between change of eGFR and change of office systolic or diastolic BP was observed. Noticeably, the relationship described between change in mGFR and change in PWV remained significant after adjustment for the change of systolic BP and the change of heart rate after 12 weeks (*r* = 0.422, *p* = 0.018). In the non-SGLT2 inhibitor based treatment group no such correlations of the changes in systolic and diastolic BP with change in PWV were observed with neither mGFR nor eGFR.

**Fig. 3:**
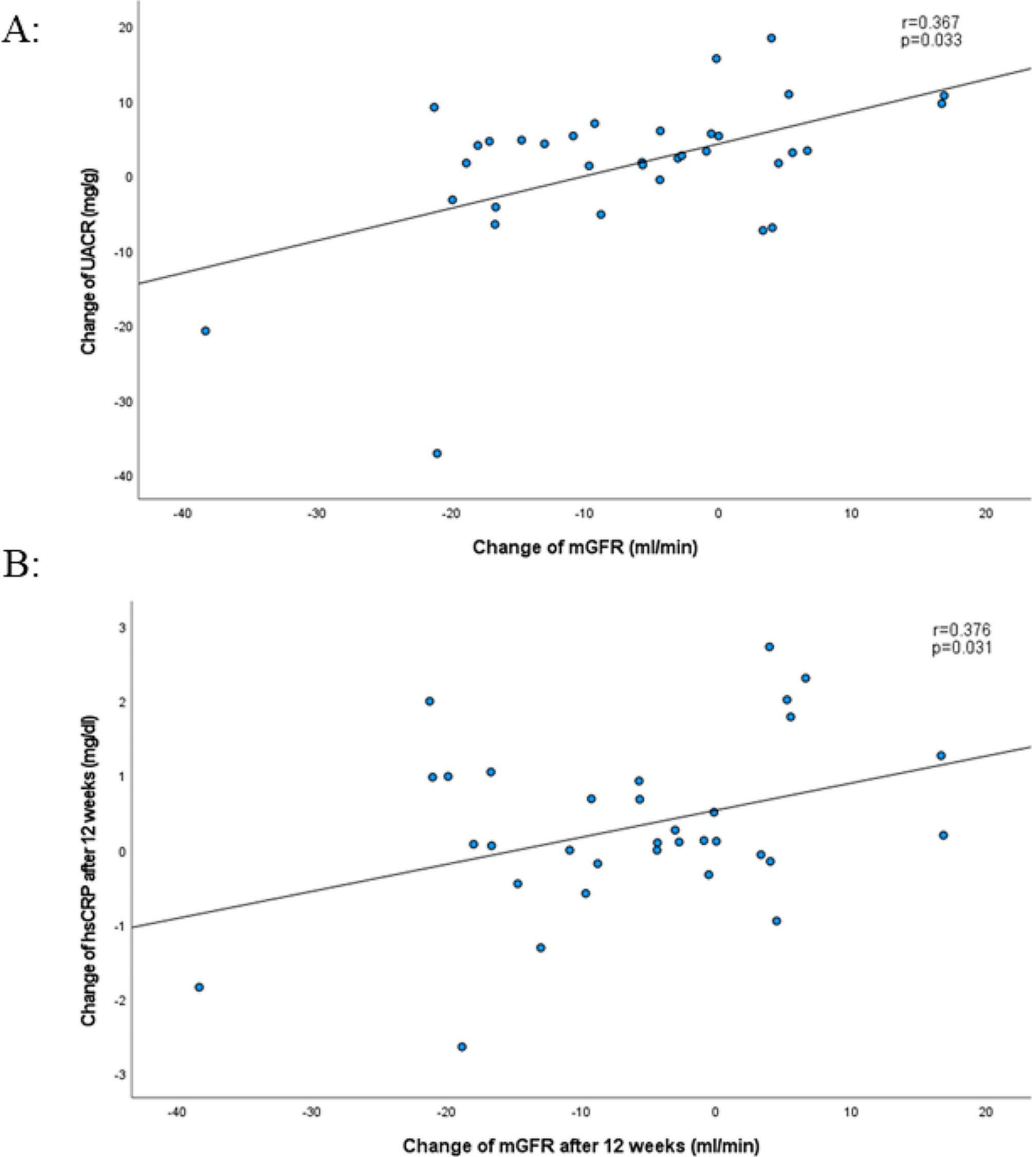
Correlation between change in in mGFR after 12 weeks of treatment and change in UACR (**A**; upper part) and change in hs-CRP (**B**; lower part) in SGLT2 inhibitor based treatment group

In addition, we observed a correlation between change in mGFR (but not with eGFR) and change in UACR and change in high sensitive C reactive protein (hs-CRP) in patients in the SGLT2 inhibitor based treatment only (*r* = 0.367, *p* = 0.033 and *r* = 0.376, *p* = 0.031, Fig. 3a and b). We did not observe any correlation between change in mGFR and change in fasting plasma glucose (*r* = 0.177, *p* = 0.317) or change in HbA1c (*r*=-0.235, *p* = 0.181). No correlation between change in eGFR and change in fasting plasma glucose (*r* = 0.025, *p* = 0.889) or change in HbA1c (*r* = 0.136, *p* = 0.443) was observed as well.

### Relationship between change in mGFR and change in eGFR

**Fig. 4:**
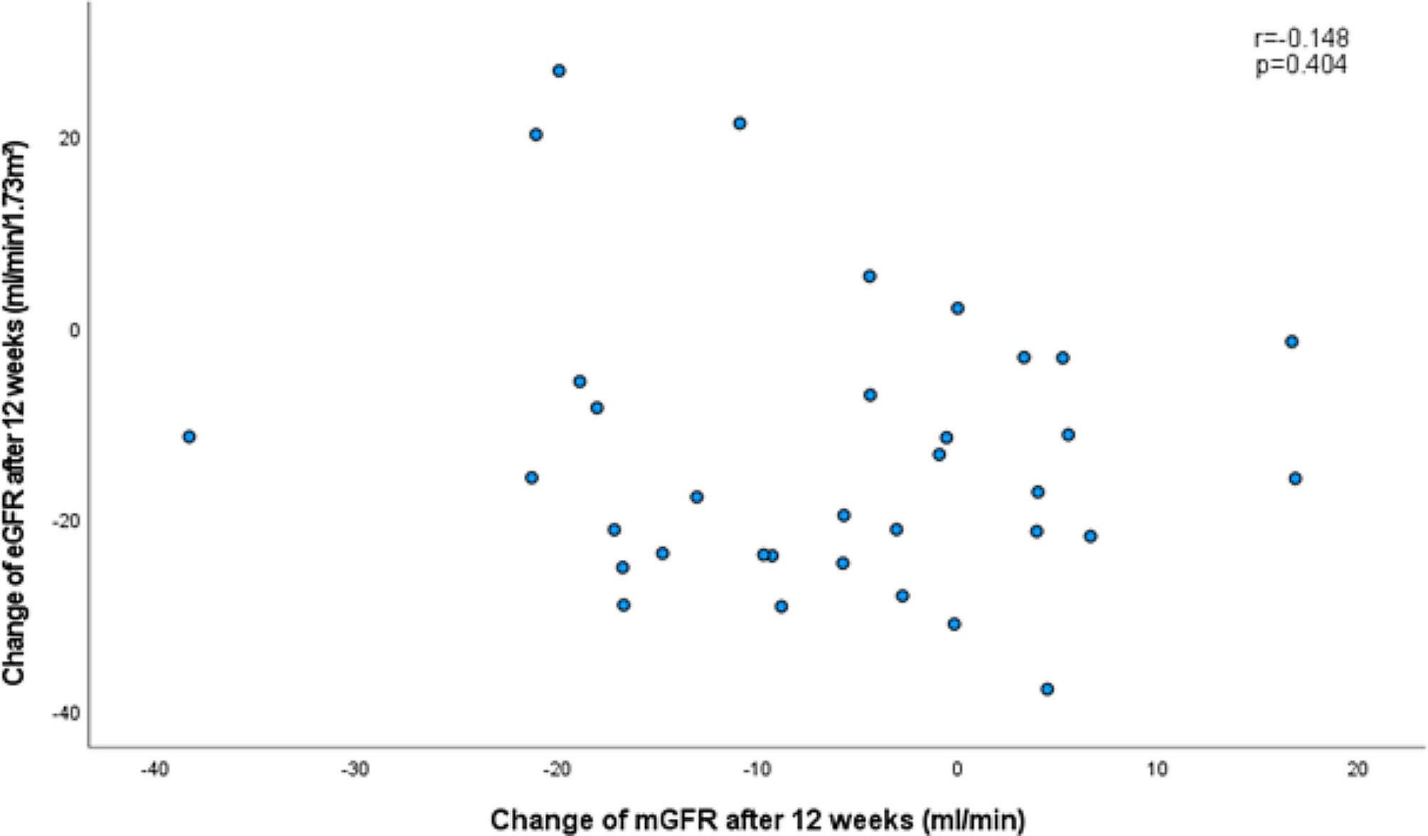
Correlation between change in mGFR and change in eGFR after 12 weeks of treatment in SGLT2 inhibitor based treatment group

**Fig. 5:**
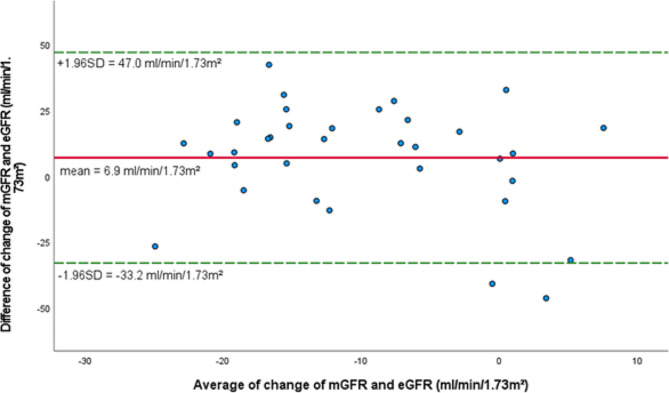
Bland-Altman Plot for the agreement between change in mGFR and change in eGFR in the SGLT2 inhibitor based treatment group

Despite a reduction of mGFR and eGFR, we observed no correlation between the change of mGFR and eGFR (*r*=-0.148, *p* = 0.404, Fig. 4). Comparing the decline in eGFR and mGFR in the SGLT2 inhibitor based treatment cohort, we found that the decline of true renal function (mGFR) tended to be lower compared to the decline of eGFR (*p* = 0.057). In accordance, the Bland-Altman plot shows poor agreement between mGFR and eGFR change with wide limits of agreement ranging from − 33.2 to + 47.0 ml/min/1.73 m² (Fig. 5).

## Discussion

SGLT2 inhibitor therapy often causes an initial decline of eGFR after starting treatment. The main finding of our study is that the initial decline in true renal function, which is defined as mGFR, with SGLT2 inhibitor treatment is linearly associated with improved vascular compliance of large arteries, thereby reflecting the pharmacologic effects of SGLT2 inhibitors both in the renal and systemic vascular bed. No such relationship was observed in the non-SGLT2 inhibitor based therapy serving as a control group in this retrospective study. Another key message of this study is that the decline in eGFR with SGLT2 inhibitor treatment does not reflect the decline in true renal function. This lack of relationship may explain also the failure to find any correlations between the change in eGFR and change in PWV. Thus, we postulate if renal function is correctly assessed, decline of GFR after starting SGLT2 inhibitor therapy is paralleled by changes of vascular compliance of large arteries in the systemic circulation.

While we observed a decline in eGFR and mGFR for both treatment arms, only patients in the SGLT2 inhibitor based treatment arm had a significant decrease in PWV from 8.2 ± 1.6 to 7.8 ± 1.5 m/s after 12 weeks of treatment. As suggested in the current guidelines of the European Society of Hypertension, PWV is known to be a unique measure of arterial stiffness and an important tool to assess vascular aging [23, 35, 36]. Two large meta-analyses revealed that this parameter is able to classify CV risk more accurately than conventional risk-based scores and that it improves CV event prediction, especially in young and middle-aged patients [26, 27]. Cherney et al. previously reported a decrease in arterial stiffness in 40 subjects with type 1 diabetes after 8 weeks of empagliflozin treatment [37]. In accordance, we previously reported beneficial effects of 12 weeks SGLT2 inhibitor based treatment regarding vascular function [20]. Noticeably, in this study we observed a correlation between the change of mGFR and the change of PWV with SGLT2 inhibitor based treatment, which demonstrates the close relationship between renal and systemic vascular bed. This correlation was only observed in the SGLT2 inhibitor based treatment group and persisted after adjustment for the change of office systolic BP and the change of heart rate after 12 weeks. Moreover, we observed a correlation between change in mGFR and change in office systolic and diastolic BP in the SGLT2 inhibitor based treatment group. Thus, the observed linear relationship between decline of mGFR and PWV indicate that decline of mGFR reflects also vascular changes in the systemic circulation exerted by SGLT2 inhibitors, and the magnitude of the initial decline of renal function is therefore of clinical relevance indicative of the magnitude of improved arterial stiffness.

In accordance, the DAPA-HF results showed an association between the initial decline in eGFR after dapagliflozin treatment and better CV outcome [11]. Compared to patients without initial eGFR decline, patients with an initial eGFR decline also had a slower long term eGFR decline [11]. Van Bommel et al. previously reported a reduction in pulse pressure in patients with T2D after 12 weeks of dapagliflozin treatment and a correlation between the reduction in pulse pressure and the reduction in mGFR [38]. Similar to PWV, pulse pressure is an indirect marker of arterial stiffness. Noticeably, in our study we observed the association between the change of mGFR and improvement of arterial stiffness independent of BP changes. In contradiction to these results a post hoc analysis of the EMPA-REG OUTCOME trial could not find any impact of the initial eGFR dip and the treatment effect of empagliflozin on CV outcome [14]. 

In accordance to our main results, we observed a correlation between change in mGFR and change in UACR in the SGLT2 inhibitor based treatment group. Increased urinary albumin excretion is considered as a marker of impaired permeability of the endothelium in the renal circulation. Likewise, transcapillary escape of radioactively marked albumin has been observed in the systemic circulation [39], thus suggesting that increased albumin escape from the blood stream (which can be easily measured in the renal vascular bed by UACR) is a marker not restricted to indicate renal prognosis. In several prospective clinical studies, albuminuria has been found to be an independent risk marker for CV events in various CV high risk populations, including T2D [40–45]. Our finding regarding the relationship of change in UACR and change in mGFR in the SGLT2 inhibitor based treatment group again support the link between the renal and systemic vascular bed.

Porrini E. et al. previously discussed the reliability of eGFR equations and concluded them to be unreliable tools to assess renal function in individual patients [46]. In accordance, we did not observe any correlation between the change of eGFR and mGFR after initiating SGLT2 inhibitor based treatment. Similarly, we have shown in a study with 190 T2D patients in the early stage of their disease that change of renal function after various short-term pharmacological intervention is not accurately and precisely reflected by the change of eGFR.

There is no doubt that carefully conducted pharmacological studies documented that SGLT2 inhibitors decrease renal function in several study cohorts. The inaccuracy of eGFR to measure renal function may be dependent on the sample size of study populations. In large-scale populations there is a consistent decrease of eGFR and individual variations of eGFR may be offset by large number of patients [2, 9, 47]. Nevertheless, from a clinical perspective, eGFR may not be an appropriate parameter to assess the true change of renal function in a given patient or small study cohorts after receiving a SGLT2 inhibitor therapy, since according to our data any decline in eGFR with SGLT2 inhibitor treatment does not necessarily reflect a decline in true renal function .

In our study, we not only observed a decline in GFR in patients with SGLT2 inhibitor based treatment, but also in patients receiving the non- SGLT2 inhibitor based treatment with insulin + metformin. Our group previously reported a decrease in renal plasma flow and renal blood flow paralleled by an increase in renal vascular resistance in patients with insulin + metformin treatment, which may explain the decline in GFR in this treatment group [17]. 

### Limitations

Our study has several limitations. It is a single-center analysis with a small sample size due to the forced by the lack of availability of inulin due to its withdrawal from the market. However, it is a prespecified analysis and in contrast to the large controlled randomized trials [2, 9, 11, 14], we measured GFR in our patients with the “gold standard”, which is a reliable tool to assess true renal function. Patients were randomized to receive either empagliflozin and linagliptin or metformin and insulin. For our present analysis this combination treatment is a limitation, since it is not possible to attribute the association between vascular improvement and mGFR decline only to empagliflozin treatment. However, we previously showed that linagliptin treatment does not cause any decline in mGFR or eGFR or any other renal hemodynamic parameter [48]. Thus, we believe that the GFR decline in our trial and its correlation to the change in PWV is attributed to the initiation of empagliflozin treatment. Our study cohort include only patients with T2D and our findings cannot be necessarily transferred to other cohorts like patients with type 1 diabetes, chronic kidney disease or heart failure.

## Conclusion

In conclusion, we found that initial decline in true renal function with SGLT2 inhibitor based treatment goes in parallel with improved vascular stiffness of large arteries, thereby reflecting the pharmacologic effects of SGLT2 inhibitors both in the renal and systemic vascular bed. Thus, our data indicate that decline of renal function reflects also changes in vascular function in the systemic circulation, i.e. improvement of arterial stiffness. According to our observations, eGFR may not be an appropriate parameter to assess the true change in renal function after receiving SGLT2 inhibitor based treatment in an individual patient and potentially may lead to the inappropriate decision to discontinue SGLT2 inhibitor therapy.

## Data Availability

The datasets used and/or analyzed during the current study are available from the corresponding author on reasonable request.

## Abbreviations

SGLT2: Sodium–glucose–cotransporter–2
CV: cardiovascular
GFR: glomerular filtration rate
mGFR: measured GFR
eGFR: estimated GFR
T2D: type 2 diabetes
BP: blood pressure
PWV: pulse wave velocity
U: units
UACR: urinary albumin to creatinine ratio
BMI: body–mass–index
CKD: EPI–Chronic Kidney Disease Epidemiology Collaboration
SD: standard deviation
Hs: CRP–high sensitive C reactive protein
HbA1c: glycated haemoglobin
